# Methods for estimating insulin resistance from untargeted metabolomics data

**DOI:** 10.1007/s11306-023-02035-5

**Published:** 2023-08-09

**Authors:** Fang-Chi Hsu, Nicholette D. Palmer, Shyh-Huei Chen, Maggie C. Y. Ng, Mark O. Goodarzi, Jerome I. Rotter, Lynne E. Wagenknecht, Michael P. Bancks, Richard N. Bergman, Donald W. Bowden

**Affiliations:** 1 Department of Biostatistics and Data Science, Wake Forest University School of Medicine, Winston-Salem, NC USA; 2 Department of Biochemistry, Wake Forest University School of Medicine, 1 Medical Center Boulevard, Winston-Salem, NC 27157 USA; 3 Vanderbilt Genetics Institute, Division of Genetic Medicine, Vanderbilt University Medical Center, Nashville, TN USA; 4 Division of Endocrinology, Diabetes and Metabolism, Department of Medicine, Cedars-Sinai Medical Center, Los Angeles, CA USA; 5 The Institute for Translational Genomics and Population Sciences, Department of Pediatrics, The Lundquist Institute for Biomedical Innovation at Harbor-UCLA Medical Center, Torrance, CA USA; 6 Division of Public Health Sciences, Wake Forest University School of Medicine, Winston-Salem, NC USA; 7 Department of Epidemiology and Prevention, Wake Forest University School of Medicine, Winston-Salem, NC 27157 USA; 8 Diabetes and Obesity Research Institute, Cedars-Sinai Medical Center, Los Angeles, CA USA

**Keywords:** Insulin resistance, Metabolomics, Machine learning, LASSO, Elastic net

## Abstract

**Context:**

Insulin resistance is associated with multiple complex diseases; however, precise measures of insulin resistance are invasive, expensive, and time-consuming.

**Objective:**

Develop estimation models for measures of insulin resistance, including insulin sensitivity index (SI) and homeostatic model assessment of insulin resistance (HOMA-IR) from metabolomics data.

**Design:**

Insulin Resistance Atherosclerosis Family Study (IRASFS).

**Setting:**

Community based.

**Participants:**

Mexican Americans (MA) and African Americans (AA).

**Main outcome:**

Estimation models for measures of insulin resistance, i.e. SI and HOMA-IR.

**Results:**

Least Absolute Shrinkage and Selection Operator (LASSO) and Elastic Net regression were used to build insulin resistance estimation models from 1274 metabolites combined with clinical data, e.g. age, sex, body mass index (BMI). Metabolite data were transformed using three approaches, i.e. inverse normal transformation, standardization, and Box Cox transformation. The analysis was performed in one MA recruitment site (San Luis Valley, Colorado (SLV); N = 450) and tested in another MA recruitment site (San Antonio, Texas (SA); N = 473). In addition, the two MA recruitment sites were combined and estimation models tested in the AA recruitment sample (Los Angeles, California; N = 495). Estimated and empiric SI were correlated in the SA (r^2^ = 0.77) and AA (r^2^ = 0.74) testing datasets. Further, estimated and empiric SI were consistently associated with BMI, low-density lipoprotein cholesterol (LDL), and triglycerides. We applied similar approaches to estimate HOMA-IR with similar results.

**Conclusions:**

We have developed a method for estimating insulin resistance with metabolomics data that has the potential for application to a wide range of biomedical studies and conditions.

**Supplementary Information:**

The online version contains supplementary material available at 10.1007/s11306-023-02035-5.

## Introduction

Insulin resistance is a central feature of glucose homeostasis and overall metabolic health. Specifically, it has a well-known central role in type 2 diabetes (T2D), is a common and early feature of Alzheimer’s disease (AD) (Talbot et al. 2012; Arnold et al. 2018), and is widely accepted as a risk factor for cardiovascular disease (CVD), both heart (Laakso & Kuusisto 2014)and stroke (Deng et al. 2017). Insulin resistance is definitively linked to disorders such as obesity, e.g. (Boden 2011), dyslipidemia, non-alcoholic fatty liver disease (NAFLD; (Barchetta et al. 2022)), and hypertension (McGill et al. 2009). More recently, insulin resistance has been linked to heart failure (Riehle & Abel 2016), psychiatric disorders (Kowalchuk et al. 2019), diabetic kidney disease (Spoto et al. 2016), and sleep (Mesarwi et al. 2013)with emerging evidence that diabetes, and thus possibly insulin resistance, is a significant risk factor in the development of numerous cancers (Ohkuma et al. 2018). These associations span major areas of biomedical significance; yet, the magnitude of risk imparted by insulin resistance is largely unknown (Laakso & Kuusisto 2014).

Outside of the focused area of diabetes research, insulin resistance is infrequently measured and rarely measured well. Insulin resistance is measured in a wide variety of ways in human subjects (Muniyappa et al. 2008; Pisprasert et al. 2013). Among these methods, the homeostatic model assessment of insulin resistance (HOMA-IR; a basal, steady state measure) (Matthews et al. 1985) has been widely used in clinical and epidemiological studies. HOMA-IR is relatively inexpensive since it is derived from measurement of glucose and insulin in a fasting blood sample. Despite its convenience, HOMA-IR is widely regarded to have significant limitations (Pisprasert et al. 2013; Wallace et al. 2004; Thompson et al. 2014) and can even lead to different conclusions compared to other insulin resistance measures (Reaven 2013). Broadly speaking, it is widely accepted that methods such as the hyperinsulinemic-euglycemic clamp (Defronzo 1979) or the frequently sampled intravenous glucose tolerance test [FSIGT; (Bergman 1989)] are more desirable. In contrast to HOMA-IR, these methods are invasive, expensive, and have to be performed in an environment such as a clinical research unit. Consequently, they are rarely used in studies outside of diabetes. Moreover, different measurement methods do not lead to identical results. For example, correlations of the insulin sensitivity index (SI from the FSIGT) and HOMA-IR are − 0.73 and − 0.62 for Mexican Americans and African Americans, respectively, in the Insulin Resistance Atherosclerosis Family Study (IRASFS) (Wagenknecht et al. 2003; Palmer et al. 2016).

A challenge to better understanding the contribution of insulin resistance to disease is the lack of estimation methodology that is not dependent on invasive testing and, ideally, would readily calculate multiple measures of insulin resistance from a single blood sample. We have built upon our prior observations that data from contemporary large-scale untargeted metabolomic analyses can account for a substantial amount of the inter-individual variation in measures of insulin resistance (Palmer et al. 2018). This suggests that we can develop estimation methods combining metabolomics and conventional clinical data, e.g. age, sex, body mass index (BMI). Here we make use of a “big data” resource, untargeted metabolomics, to build statistical models that accurately estimate multiple measures of insulin resistance using data from an extensively phenotyped population-based cohort.

## Materials and methods

### Study population

The Insulin Resistance Atherosclerosis Family Study (IRASFS) is a population-based study designed to investigate the genetic and environmental basis of glucose homeostasis and visceral adiposity. The study design, recruitment, and phenotyping have been described (Henkin et al. 2003). Specifically, Mexican Americans (MA) were recruited from clinical centers in San Luis Valley, CO (SLV) and San Antonio, TX (SA). African Americans (AA) were recruited from a single clinical center in Los Angeles, CA (LA). All sites performed identical ascertainment and phenotyping. The study protocol was approved by the Institutional Review Board of each participating clinical and analysis site. All participants provided their written informed consent.

### Phenotyping

A clinical examination was performed that included an interview, a frequently sampled intravenous glucose tolerance test (FSIGT) for subjects without T2D, anthropometric measurements, and blood collection. A dynamic measure of glucose homeostasis, the insulin sensitivity index (SI), was calculated from the FSIGT using the reduced sampling protocol (Bergman et al. 1979; Steil et al. 1993) with mathematical modeling methods (MINMOD) (Pacini & Bergman 1986). HOMA-IR was modeled from fasting glucose and insulin measures using the updated HOMA model (Levy et al. 1998).

### Metabolomics

Metabolite profiling of IRASFS fasting plasma samples stored at − 80 °C since baseline collection from 1999 to 2002 was performed at Metabolon, Inc. (Durham, North Carolina) with detection and quantification of 1274 metabolites using untargeted liquid chromatography-mass spectrometry (LC–MS; DiscoveryHD4 panel). This panel identifies known chemical compounds (N = 912) and uncharacterized compounds that do not currently have chemical standards (N = 362). Prior to receipt, data were block corrected for run day, normalized by batch, and missing data by metabolite was imputed to the minimum. A detailed description of quality control and data preparation has been reported (Palmer et al. 2018). Upon receipt, sample-level analysis revealed an excess of metabolite outliers (± 4 SD; N = 134) for a single sample which was subsequently removed from analysis. Individual metabolite values were winsorized at 1 and 99% to reduce the effect of spurious outliers.

### Statistical analysis

Our goal was to develop estimation models for measures of insulin resistance, i.e. SI and HOMA-IR, from metabolomic data combined with conventional clinical data, i.e. age, sex, and BMI. Three trait transformations were implemented, i.e. inverse normal transformation (INT), standardization, and Box Cox transformation, to account for distribution differences among metabolites and these were subsequently tested to optimize estimation. INT is a rank-based approach in which all metabolites were normally distributed. Standardization is to rescale the metabolite to have a mean of zero and a standard deviation of one. The Box Cox transformation identifies the best transformation for each metabolite independently. Further, two regularized regression methods, i.e. Least Absolute Shrinkage and Selection Operator (LASSO) (Tibshirani 1996) and Elastic Net (Zou & Hastie 2005), were fit to build the estimation models. The IRASFS MA sample was divided by the two recruitment sites, SLV and SA, with approximately equal sample sizes for training and testing. In SLV (training dataset), metabolomic data were fitted in the regularized regression model where empiric SI was the outcome measure. A 10-fold cross-validation was used to select the best elastic net mixing parameter (alpha = 0.25, 0.5, 0.75, or 1). The selected metabolites from the regularized regression model as well as age, sex, and BMI were then fit in the mixed effects model, with kinship covariance structure and random intercept to account for family structure, to estimate SI in the SLV cohort. The model can be written as below,

$$Y_{{{\text{ij}}}} {\text{ }} = {\text{ }}\beta _{{\text{0}}} {\text{ }} + {\text{ }}\beta _{{\text{1}}} {\text{ }}Age_{{{\text{ij}}}} {\text{ }} + {\text{ }}\beta _{{\text{2}}} {\text{ }}Sex_{{{\text{ij}}}} {\text{ }} + {\text{ }}\beta _{{\text{3}}} {\text{ }}BMI_{{{\text{ij}}}} {\text{ }} + {\text{ }}\beta _{{\text{4}}} {\text{ }}Metabolite_{{{\text{1ij}}}} {\text{ }} + {\text{ }} \ldots {\text{ }} + {\text{ }}\beta _{{\text{q+3}}} {\text{ }}Metabolite_{{{\text{qij}}}} {\text{ }} + {\text{ }}\varepsilon _{{{\text{ij,}}}}$$

where Y_ij_ is log transformed SI or HOMA-IR measured on the *i*^th^ family in the *j*^th^ individual, β_1_,…,β_q+3_ are the regression coefficients, and ε_ij_ is the error term for *i*^th^ family in the *j*^th^ individual. We then directly used the regression coefficient estimates from this model to estimate the log transformed SI or HOMA-IR in SA (testing dataset). The estimated measure is calculated as.

$$\hat{Y}_{{{\text{ij}}}} \, = \,\widehat{\beta }_{{{\text{0}}\,}} \, + \,\widehat{\beta }_{{\text{1}}} \,Age_{{{\text{ij}}\,\,}} + \,\widehat{\beta }_{{\text{2}}} \,sex_{{{\text{ij}}}} \, + \,\widehat{\beta }_{{\text{3}}} \,BMI_{{{\text{ij}}}} \, + \,\widehat{\beta }_{{\text{4}}} \,Metabolite_{{{\text{1ij}}}} \, + \,...\, + \,\widehat{\beta }_{{\text{q+3}}} \,Metabolite_{{{\text{qij}}}} \,$$

The correlation between estimated and empiric measures was calculated using Spearman’s rank correlation coefficient. This model is our full model. For comparison, we also fit three nested models using the mixed effects models in SLV: (1) age and sex, (2) age, sex, and BMI, and (3) age, sex, and associated metabolites (Palmer et al. 2018). The correlation coefficients between estimated and empiric measures for these models in SA were calculated. Additionally, we built the estimation models using MA (combined SLV and SA) data for these two regularized regression models and applied them to AA. Different subsets of metabolites were selected. To test the validity of estimated measures, we tested the correlation of estimated SI and empiric HOMA-IR, estimated HOMA-IR and empiric SI, and estimated SI and HOMA-IR measures with BMI (estimated without BMI in the model), low-density lipoprotein cholesterol (LDL), and triglycerides using Spearman’s rank correlation coefficients.

## Results

Demographic and clinical characteristics of the study sample are shown in Table 1. The study sample comprised data from three clinical sites, including 450 MAs from 30 pedigrees in San Luis Valley, CO (SLV), 473 MAs from 58 pedigrees in San Antonio, TX (SA), and 495 AAs from 42 pedigrees in Los Angeles, CA (LA). In the combined sample, 59% were female with a mean ± standard deviation age of 39.9 ± 13.1 years, BMI of 28.7 ± 6.1 kg/m^2^, SI of 2.0 ± 1.7 × 10^−4^ min/µU/mL, and HOMA-IR of 1.6 ± 1.0. SA and LA had a slightly higher BMI compared to SLV. SLV had the higher dynamic measure of insulin resistance, i.e. SI, and lower basal measure, i.e. HOMA-IR, compared to SA and LA. LA had a lower SI than SA and SLV. HOMA-IR for LA was similar to that observed in SLV.

**Table 1:** Demographic and clinical characteristics of the IRASFS cohort

Variables	Overall (n = 1418)	SLV (n = 450)	SA (n = 473)	LA (n = 495)
Age, years	39.9 ± 13.1	40.4 ± 12.7	38.6 ± 13.3	40.7 ± 13.3
Female, %	832 (59%)	265 (59%)	282 (60%)	285 (58%)
BMI, kg/m^2^	28.7 ± 6.1	27.1 ± 5.1	29.5 ± 6.1	29.4 ± 6.6
LDL, mg/dL	109.8 ± 30.8	107.5 ± 29.2	108.9 ± 29.3	112.8 ± 33.2
Triglycerides, mg/dL	101.9 ± 76.2	110.7 ± 70.1	123.4 ± 93.4	73.3 ± 50.2
SI, x 10^−4^ min/µU/mL	2.0 ± 1.7, 1.6 (0.9, 2.6)	2.3 ± 1.8, 1.9 (1.0, 3.3)	2.0 ± 1.9, 1.5 (0.8, 2.6)	1.6 ± 1.2, 1.4 (0.8, 2.3)
HOMA-IR	1.6 ± 1.0, 1.3 (0.9, 2.1)	1.5 ± 0.9, 1.2 (0.9, 1.9)	1.8 ± 1.2, 1.5 (1.0, 2.2)	1.6 ± 1.0, 1.3 (0.9, 2.1)

Values represent the mean ± standard deviation unless otherwise noted. For SI, HOMA-IR, mean ± standard deviation, median (Q1, Q3) are presented

Among the 1274 metabolites, in SLV 47 INT, 35 standardized, and 48 Box Cox transformed metabolites were selected for estimating log SI using LASSO and 59 INT, 59 standardized, and 68 Box Cox transformed metabolites were selected using Elastic Net for models estimating SI. Among the selected metabolites, 60–83% were commonly selected using the three transformations and 66–94% were selected using at least two transformations. Given the same transformation, 58–100% were selected by both LASSO and Elastic Net. The list of selected metabolites is provided in **ST1**.(Supplement. https://figshare.com/s/4173e38658bb11b3ef8c).

Table 2 shows the results from LASSO and Elastic Net models, comparing the three metabolite transformations for the full model (Model 3L: age, sex, BMI and metabolites selected using LASSO and Model 3E: age, sex, BMI and metabolites selected using Elastic Net) and the three nested models (Model 0, age and sex; Model 1, age, sex and BMI; Model 2L, age, sex and metabolites selected using LASSO; and Model 2E, age, sex and metabolites selected using Elastic Net) with appropriate covariate adjustments. Overall, transformations had a minimal impact model performance. For the LASSO model, including metabolites in the prediction model with age, sex, and BMI improved estimation and resulted in correlations of 0.76–0.78 between estimated and empiric SI (SLV predicting SA; Model 3L). Similar results were observed with the Elastic Net models (Model 3E), i.e. correlations of 0.75–0.78 between estimated and empiric SI.

**Table 2:** Spearman’s rank correlation coefficients between empiric and estimated log SI and log HOMA-IR and between empiric log HOMA-IR and estimated log HOMA-IR

Training	Testing	Metabolite	Model 0	Model 1	LASSO		Elastic Net	
		Transformation			Model 2L	Model 3L	Model 2E	Model 3E
The correlation between empiric log SI and estimated log SI								
SLV	SA	INT	0.24	0.61	0.78	0.78	0.76	0.76
		Standardized	0.24	0.61	0.76	0.77	0.77	0.78
		Box cox	0.24	0.61	0.76	0.77	0.74	0.75
MA	AA	INT	0.27	0.54	0.72	0.72	0.72	0.73
		Standardized	0.27	0.54	0.73	0.74	0.73	0.74
		Box cox	0.27	0.54	0.73	0.73	0.73	0.73
The correlation between empiric log HOMA-IR and estimated log SI								
SLV	SA	INT	− 0.12*	− 0.60	− 0.65	− 0.66	− 0.65	− 0.66
		Standardized	− 0.12*	− 0.60	− 0.64	− 0.66	− 0.64	− 0.66
		Box cox	− 0.12*	− 0.60	− 0.63	− 0.65	− 0.64	− 0.66
MA	AA	INT	− 0.08*	− 0.48	− 0.60	− 0.61	− 0.60	− 0.61
		Standardized	− 0.08*	− 0.48	− 0.59	− 0.61	− 0.60	− 0.60
		Box cox	− 0.08*	− 0.48	− 0.61	− 0.63	− 0.60	− 0.62
The correlation between empiric log HOMA-IR and estimated log HOMA-IR								
SLV	SA	INT	− 0.11*	0.58	0.71	0.72	0.71	0.72
		Standardized	− 0.11*	0.58	0.70	0.71	0.70	0.71
		Box cox	− 0.11*	0.58	0.71	0.72	0.71	0.72
MA	AA	INT	0.03*	0.49	0.64	0.66	0.66	0.67
		Standardized	0.03*	0.49	0.66	0.68	0.66	0.68
		Box cox	0.03*	0.49	0.66	0.67	0.66	0.67

*SLV* San Luis Valley; *SA* San Antonio; *MA* Mexican American; *AA* African American; *INT* inverse normal transformation; Model 0: adjusted for age and gender; Model 1: adjusted for age, gender, and BMI; Model 2L: adjusted for age, gender, metabolites selected using LASSO; Model 3L: adjusted for age, gender, BMI, and metabolites selected using LASSO; Model 2E: adjusted for age, gender, metabolites selected using Elastic Net; Model 3E: adjusted for age, gender, BMI, and metabolites selected using Elastic Net. *All p-values are < 0.001 except the following: for the Model 0 in the correlation between empiric log HOMA-IR and estimated log SI: from SLV to SA, p-value = 0.010; from MA to AA, p-value = 0.07, and for the Model 0 in the correlation between empiric log HOMA-IR and estimated log HOMA-IR: from SLV to SA, p-value = 0.016; from MA to AA, p-value = 0.34

Next, we combined the MA cohorts and tested the MA estimation models for log SI in AAs. In MA, 67 INT, 70 standardized, and 51 Box Cox transformed metabolites were selected for log SI using LASSO and 84 INT, 82 standardized, and 64 Box Cox transformed metabolites were selected using Elastic Net [**ST2. **(Supplement. https://figshare.com/s/4173e38658bb11b3ef8c)]. As seen in Table2, cross-ethnic validation worked well with correlation between empiric and estimated log SI for both LASSO (Model 3L; correlation from 0.72 to 0.74) and Elastic Net (Model 3E; correlation 0.73–0.74).

Finally, the Spearman’s rank correlation coefficient was used to compare estimated log SI with empiric log HOMA-IR (Table 2), BMI, LDL, and triglycerides (Table 3). Estimated log SI was negatively correlated with log HOMA-IR (all p-values < 0.001) in Model 3 for both LASSO and Elastic Net, for both of the two training and testing sets across the three metabolite transformations. BMI and triglycerides were negatively associated with empiric and estimated log SI in SA (all p-values < 0.001). BMI, LDL, and triglycerides were negatively associated with empiric and estimated log SI in AA (all p-values < 0.001). Effect sizes were comparable for both the estimated and empiric SI measures. Results for the LASSO and Elastic Net models were broadly similar.

**Table 3:** Spearman’s rank correlation coefficients of BMI, LDL and triglycerides with empiric and estimated log SI and log HOMA-IR. using inverse normal transformation of the metabolites

Variable	Training	Testing	BMI^a^	LDL^b^	Triglycerides^b^
Empiric SI		SA	− 0.58, < 0.001	− 0.05, 0.15	− 0.43, < 0.001
		AA	− 0.53, < 0.001	− 0.28, < 0.001	− 0.34, < 0.001
Estimated SI	SLV	SA	− 0.66, < 0.001	− 0.03, 0.11	− 0.49, < 0.001
(LASSO)	MA	AA	− 0.59, < 0.001	− 0.24, < 0.001	− 0.46, < 0.001
Estimated SI	SLV	SA	− 0.68, < 0.001	− 0.01, 0.23	− 0.47, < 0.001
(Elastic Net)	MA	AA	− 0.58, < 0.001	− 0.23, < 0.001	− 0.45, < 0.001
Empiric HOMA-IR		SA	0.59, < 0.001	0.06, 0.06	0.35, < 0.001
		AA	0.50, < 0.001	0.16, < 0.001	0.29, < 0.001
Estimated HOMA-IR	SLV	SA	0.63, < 0.001	0.05, 0.090	0.45, < 0.001
(LASSO)	MA	AA	0.55, < 0.001	0.26, < 0.001	0.40, < 0.001
Estimated HOMA-IR	SLV	SA	0.59, < 0.001	0.06, 0.027	0.42, < 0.001
(Elastic Net)	MA	AA	0.55, < 0.001	0.25, < 0.001	0.40, < 0.001

*SLV* San Luis Valley; *SA* San Antonio; *MA* Mexican American; *AA* African American. ^a^Estimated SI or HOMA-IR was calculated based on Model 2 (estimated based on age, gender, and metabolites), ^b^estimated SI or HOMA-IR was calculated based on Model 3 (estimated based on age, gender, BMI, and metabolites)

We applied the same approach to estimate log HOMA-IR. Among the 1274 metabolites in SLV, 24 INT, 28 standardized, and 40 Box Cox transformed metabolites were selected for log HOMA-IR using LASSO and 53 INT, 73 standardized, and 40 Box Cox transformed metabolites were selected using Elastic Net. Among the selected metabolites, 45–83% were commonly identified using the three transformations and 55–90% were identified using at least two transformations. Given the same transformation, 38–100% were selected by both LASSO and Elastic Net. The list of selected metabolites can be found in **ST3. **(Supplement. https://figshare.com/s/4173e38658bb11b3ef8c). The correlations between estimated and empiric log HOMA-IR ranged from 0.71 to 0.72 when testing the SLV estimation models using metabolites selected by LASSO or Elastic Net (Table2; Model 3L and 3E, respectively).

In MA, 55 INT, 44 standardized, and 54 Box Cox transformed metabolites were selected for log SI using LASSO and 77 INT, 50 standardized, and 54 Box Cox transformed metabolites were selected using the Elastic Net model [ST4. (Supplement. https://figshare.com/s/4173e38658bb11b3ef8c)]. When testing the MA estimation models in AAs, performance ranged from 0.66 to 0.68 for the LASSO and 0.67 to 0.68 for the Elastic Net (Table2; Model 3L and Model 3E, respectively). Overall, there was a modest decline in performance for HOMA-IR as compared to SI.

The associations between BMI, LDL, and triglycerides with empiric and estimated log HOMA-IR are presented in Table 3. BMI and triglycerides were positively associated with empiric and estimated log HOMA-IR in SA and AA (all p-values < 0.001). LDL was positively associated with empiric and estimated log HOMA-IR in AAs (all p-values < 0.001). However, LDL was not associated with empiric log HOMA-IR in SA (p = 0.06). Only estimated log HOMA-IR using LASSO validates this result. Correlations were comparable for both the estimated and empiric log HOMA-IR measures.

## Discussion

The importance of insulin resistance as a contributor to disease is widely acknowledged, as are the difficulties of measuring insulin resistance. This is reflected in the literature where methods for sampling and measuring insulin resistance continue to appear [e.g. (Eichenlaub et al. 2022)]. In our prior work, we described the association of metabolites with insulin resistance and incident type 2 diabetes (Palmer et al. 2018). Therein, we have shown that a substantial amount of the variation in measures of insulin resistance can be explained by expansive, untargeted metabolomics. An important observation from this work was that 99 metabolites were significantly associated with SI encompassing at least 28 sub-pathways. Thus, trying to explain insulin resistance by focusing on one or a few specific metabolites will likely capture only a small fraction of the variation in insulin resistance.

Building on this prior work, we used untargeted metabolomics and conventional clinical data to build statistical models that accurately estimate measures of insulin resistance. Models were derived and validated in the multiethnic IRASFS cohort. Central features of this effort were the use of a data set, IRASFS, that has both empirically measured insulin resistance (SI and HOMA-IR) and comprehensive untargeted metabolomics and application of contemporary machine learning methods for metabolite selection (LASSO and Elastic Net). Consistent with our initial conjecture, metabolomics, in combination with conventional clinical measures, demonstrated good model performance with a high correlation between estimated and empiric SI. Further, models were generalizable across race/ethnic groups, i.e. the SI estimation model from MA data worked well in AA data from IRASFS. Similarly, estimated and empiric HOMA-IR showed similar patterns of correlation across cohorts and across race/ethnic groups.

Importantly, SI, a dynamic measure of insulin resistance, and HOMA-IR, a basal measure, were both estimated from the same metabolomic data derived from a single fasting plasma sample. Comparatively, estimated SI may outperform estimated HOMA-IR as seen by the Spearman’s rank correlation coefficients. This may be attributed to the increased ability of metabolites and clinical measures to explain the variance of SI as compared to HOMA-IR. In addition, we validated estimated SI and HOMA-IR by performing comparability studies with empirically derived SI and HOMA-IR for association with biomedical traits, e.g. BMI, LDL, and triglycerides. Thus, we have developed a method for estimating insulin resistance that does not require invasive and expensive testing. In fact, the metabolomic data are derived from a single fasting plasma sample making the method applicable to almost any study of disease with a metabolic component.

As noted, there are numerous methods for measuring insulin resistance. Here we have evaluated one steady state, HOMA-IR, and one dynamic measure, SI. Our estimation models performed comparably even though the two measures range from good to moderate correlation depending on the subject sample, e.g. (Palmer et al. 2016). There is no reason that this methodology could not be applied to develop estimation models for other widely used methods for measuring insulin resistance such as the hyperinsulinemic euglycemic clamp. Applying this method to estimate multiple measures of insulin resistance from identical clinical samples may provide insight into the relationship of different insulin resistance measures and their relative value to reflect risk of disease.

LASSO (Tibshirani 1996) and Elastic Net (Zou & Hastie 2005) approaches are commonly used in molecular biology (Lacroix 2021). LASSO performs continuous shrinkage and automatic variable selection at once, shrinking some regression coefficients and setting others to 0. It avoids overfitting and does well when a small to moderate number of moderate-sized effects are available. However, LASSO selects at most n predictors when n < < p (n: number of data points; p: number of predictors). It also selects only one predictor from a group of correlated predictors and this selection is arbitrary. Elastic Net, a shrinkage and selection method, is a generalization of LASSO and often outperforms LASSO (Zou & Hastie 2005). It generalizes ridge and LASSO regularization and encourages a grouping effect in which strongly correlated predictors are more likely to be in or out of the model simultaneously. The drawback is that it is more computationally expensive. In our result, the two approaches performed comparably. Additionally, different numbers of metabolites were selected to build the prediction model for different metabolite transformations, i.e. approximately 50% of metabolites overlapped across the three transformations. However, the correlation coefficients did not change significantly. This suggests that the method is robust to the distribution of the predictors and that future work in larger cohort could identify a smaller number of key metabolites that accurately estimate insulin resistance. The result would be lower cost; thus, further streamlining application of the methods.

Further analytical development may improve the estimation models. First, we have used the most conventional clinical traits, i.e. age, sex, and BMI, since those are available for virtually all studies. Estimation may be improved by adding data for select traits with a known association with insulin resistance, e.g. blood pressure, LDL, etc. Second, measurement error was not considered, which may result in an attenuation bias when using an estimated measure in association analysis. Third, besides LASSO and Elastic Net, other approaches such as random forest or deep learning can be used to improve the estimation model. Fourth, more replication studies could be used for validation. In time, the expense and complexity of the metabolomic analysis may be reduced by targeted analysis of a much smaller number of metabolites from a blood sample. As a result, estimation models may need to be reevaluated when only a small set of metabolites are available.

In summary, we have developed methods for estimating insulin resistance in human subjects which will greatly expand the ability to include insulin resistance measurement in research and provide meaningful insight into the role of insulin resistance in disease. Our method is non-invasive and can be derived when metabolite data are available. These findings support the potential for application to a wide range of biomedical studies.

## Supplementary Information

Below is the link to the electronic supplementary material.

Supplementary material 1 (PDF 354.4 kb)

Supplementary material 2 (XLSX 62.0 kb)

## Data Availability

Original data generated and analyzed during this study are included in this published article or in the data repositories listed in References.
