# Supplementary material for: Methods for estimating insulin resistance from untargeted metabolomics data
Source: Metabolomics. 2023 Aug 9;19(8):72. doi: 10.1007/s11306-023-02035-5 (PMC10412652; doi:10.1007/s11306-023-02035-5)

Supplementary Figure Legends

Supplementary Figure 1. LASSO selection by metabolite transformation in IRASFS Mexican Americans from San Luis Valley (SLV), CO, for the insulin sensitivity index (SI).

Supplementary Figure 2. Elastic net selection by metabolite transformation in IRASFS Mexican Americans from San Luis Valley (SLV), CO, for the insulin sensitivity index (SI).

Supplementary Figure 3. LASSO selection by metabolite transformation in IRASFS Mexican Americans (MA) for the insulin sensitivity index (SI).

Supplementary Figure 4. Elastic net selection by metabolite transformation in IRASFS Mexican Americans (MA) for the insulin sensitivity index (SI).

Supplementary Figure 5. LASSO selection by metabolite transformation in IRASFS Mexican Americans from San Luis Valley (SLV), CO, for the homeostatic model assessment of insulin resistance (HOMA-IR).

Supplementary Figure 6. Elastic net selection by metabolite transformation in IRASFS Mexican Americans from San Luis Valley (SLV), CO, for the homeostatic model assessment of insulin resistance (HOMA-IR).

Supplementary Figure 7. LASSO selection by metabolite transformation in IRASFS Mexican Americans (MA) for the homeostatic model assessment of insulin resistance (HOMA-IR).

Supplementary Figure 8. Elastic net selection by metabolite transformation in IRASFS Mexican Americans (MA) for the homeostatic model assessment of insulin resistance (HOMA-IR).

Supplementary Figure 1. LASSO selection by metabolite transformation in SLV for SI.

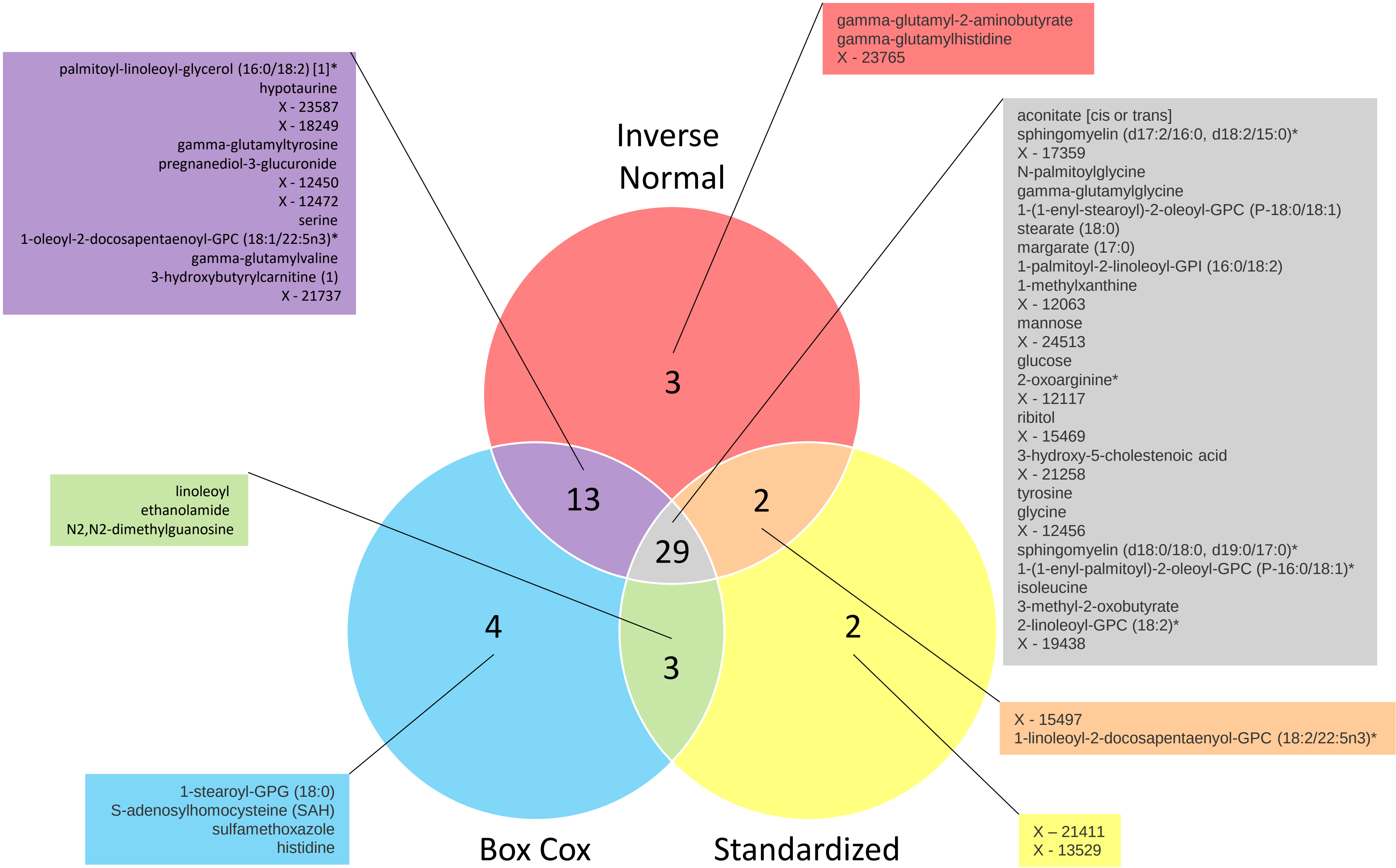

Supplementary Figure 2. Elastic Net selection by metabolite transformation in SLV for SI.

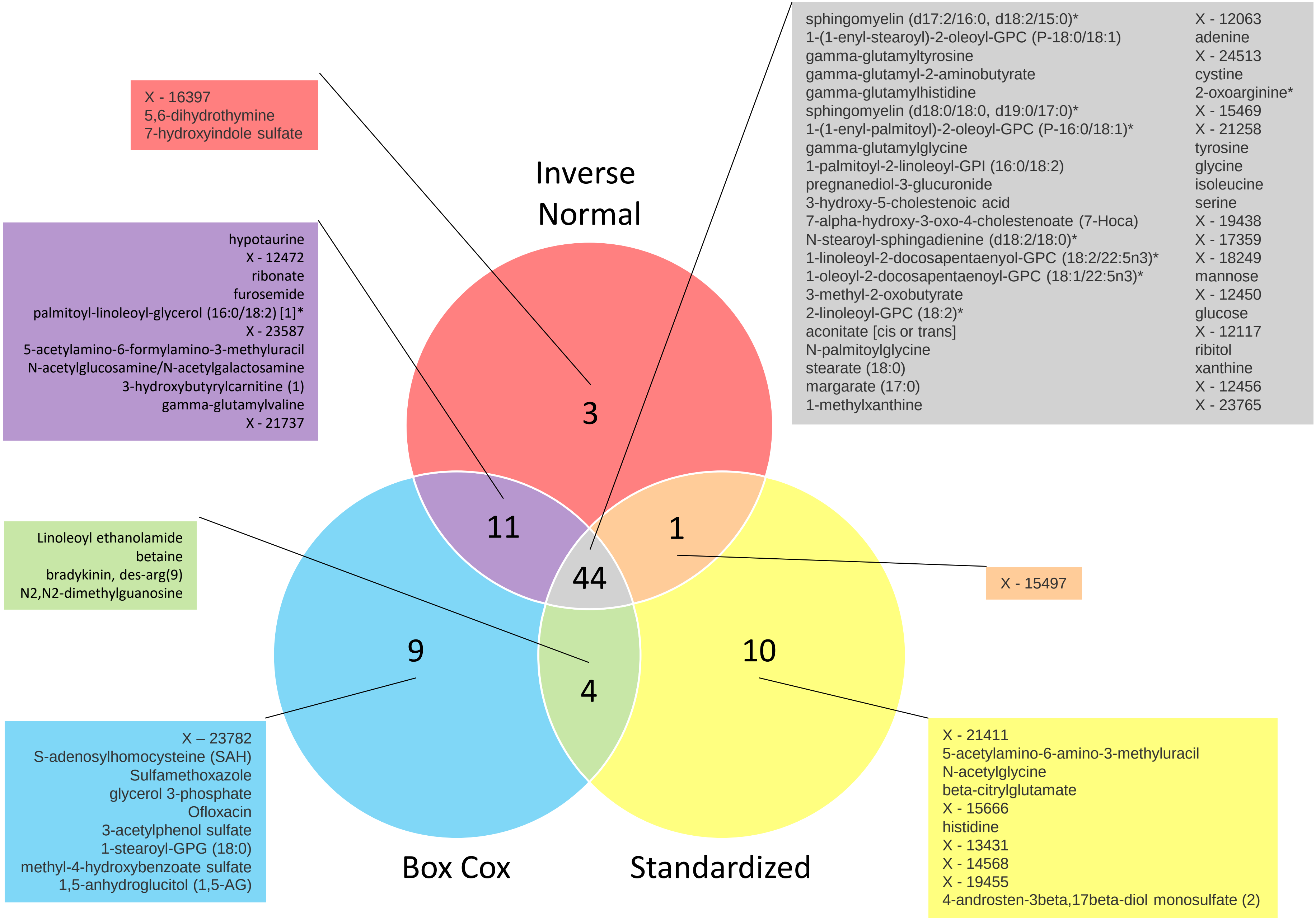

Supplementary Figure 3. LASSO selection by metabolite transformation in MA for SI.

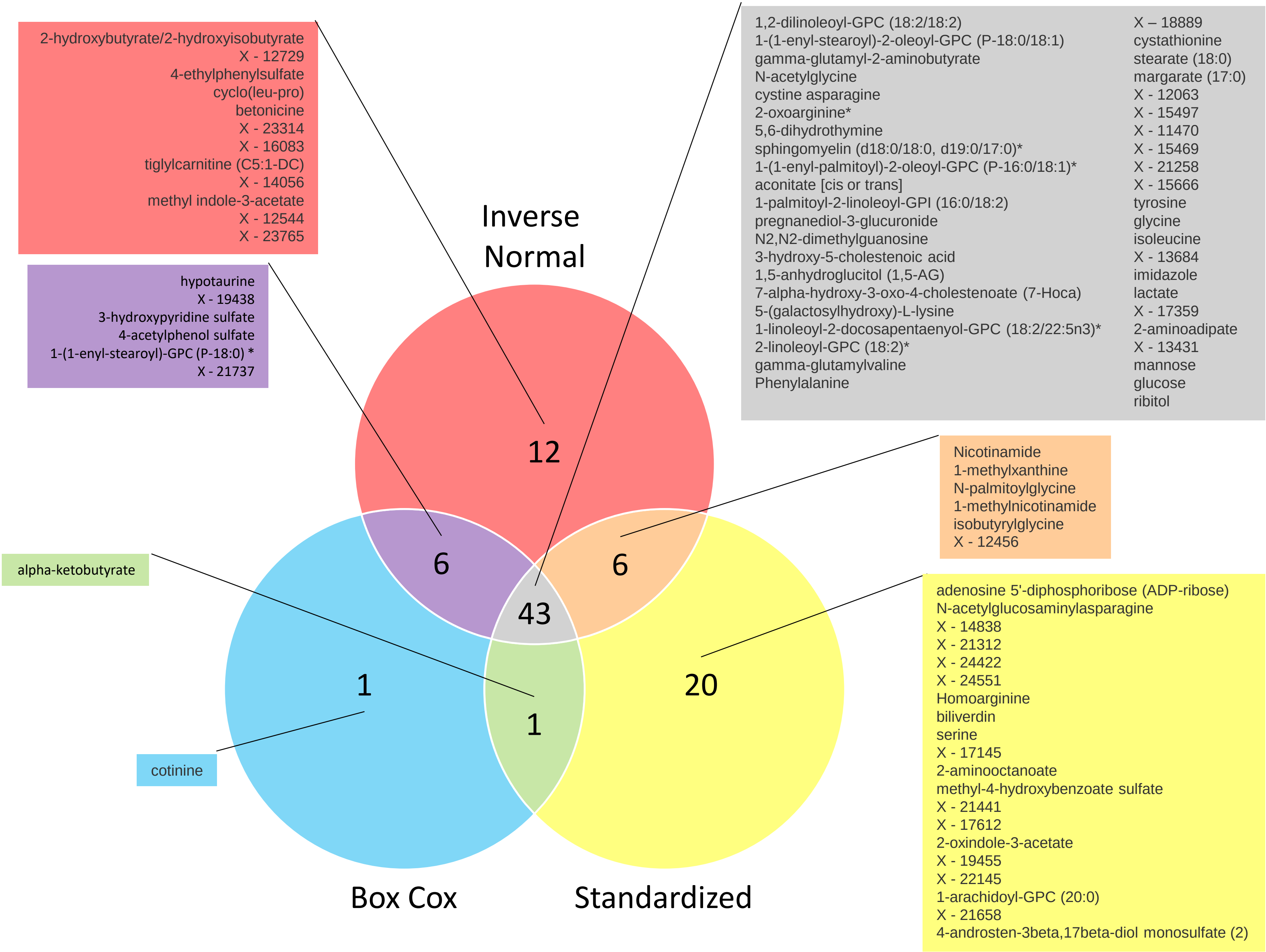

Supplementary Figure 4. Elastic Net selection by metabolite transformation in MA for SI.

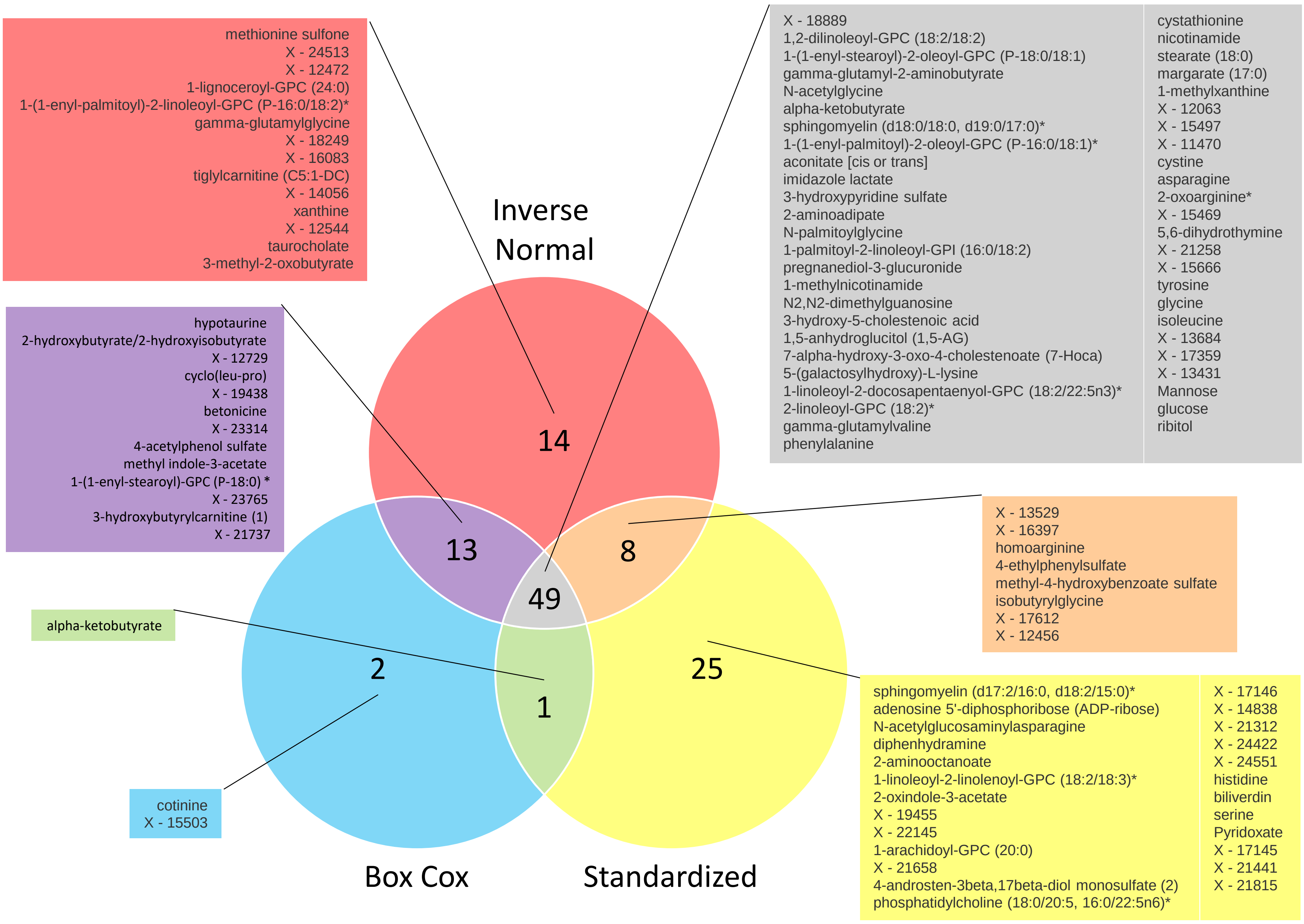

Supplementary Figure 5. LASSO selection by metabolite transformation in SLV for HOMA-IR.

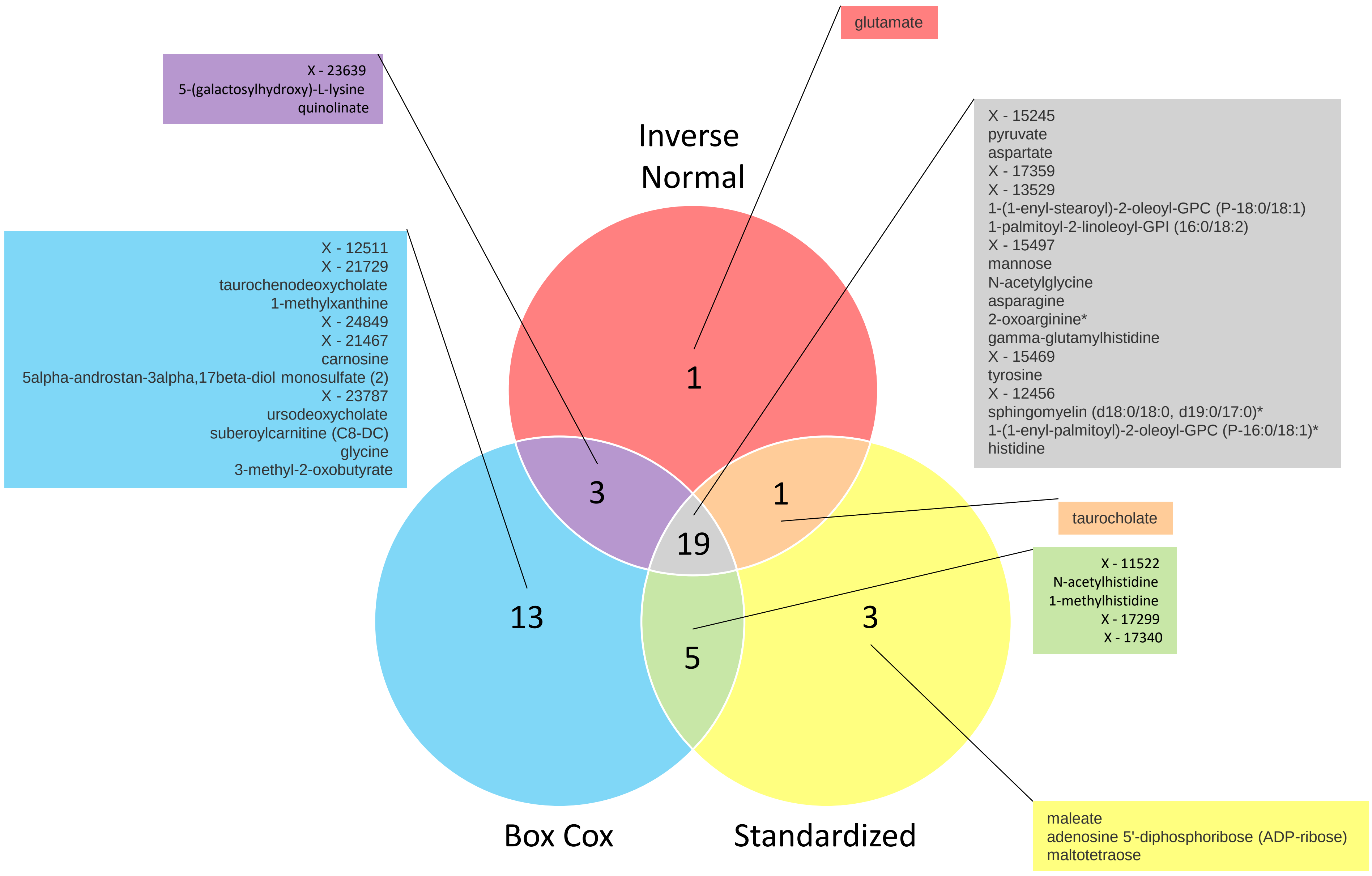

Supplementary Figure 6. Elastic Net selection by metabolite transformation in SLV for HOMA-IR.

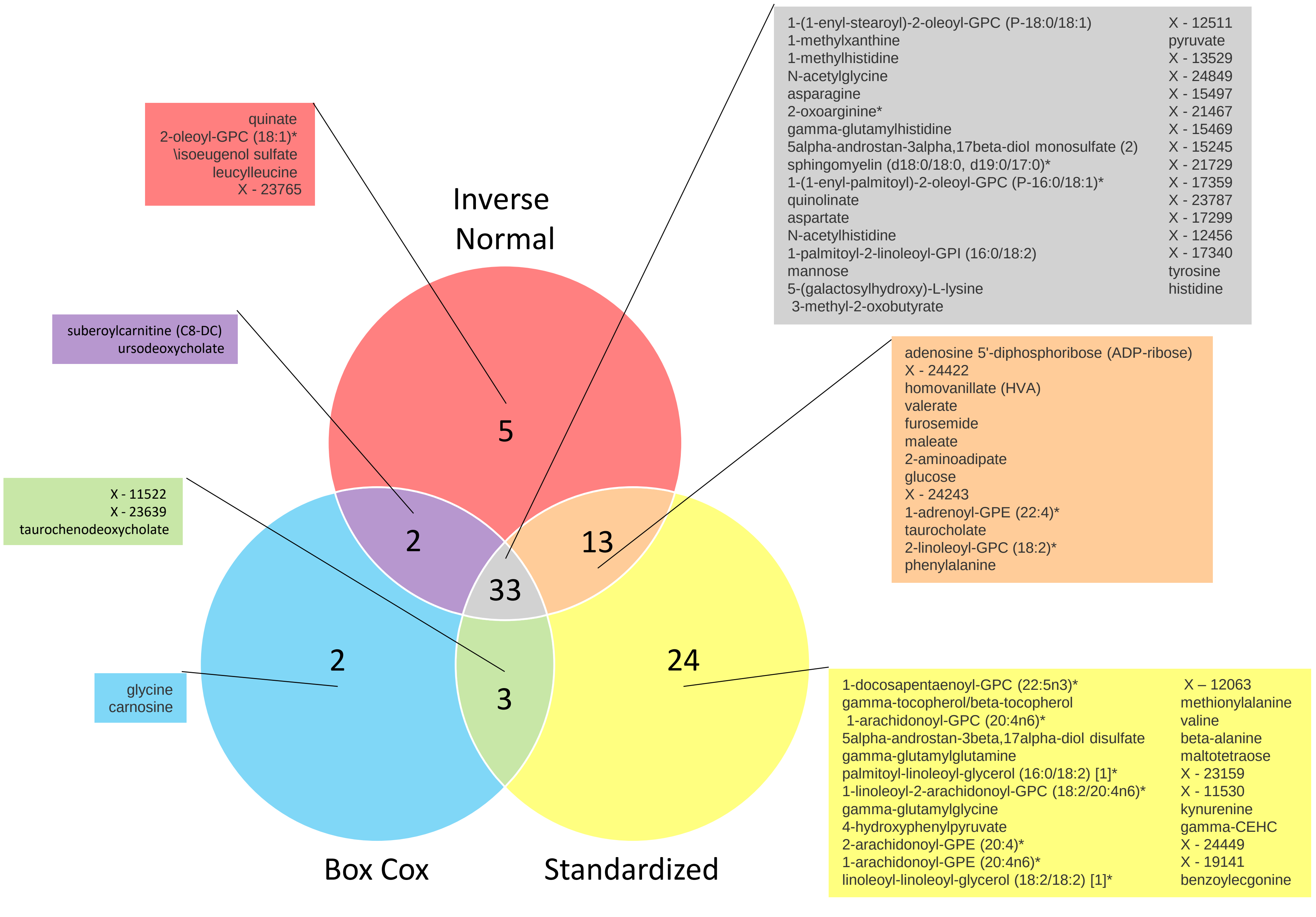

Supplementary Figure 7. LASSO selection by metabolite transformation in MA for HOMA-IR.

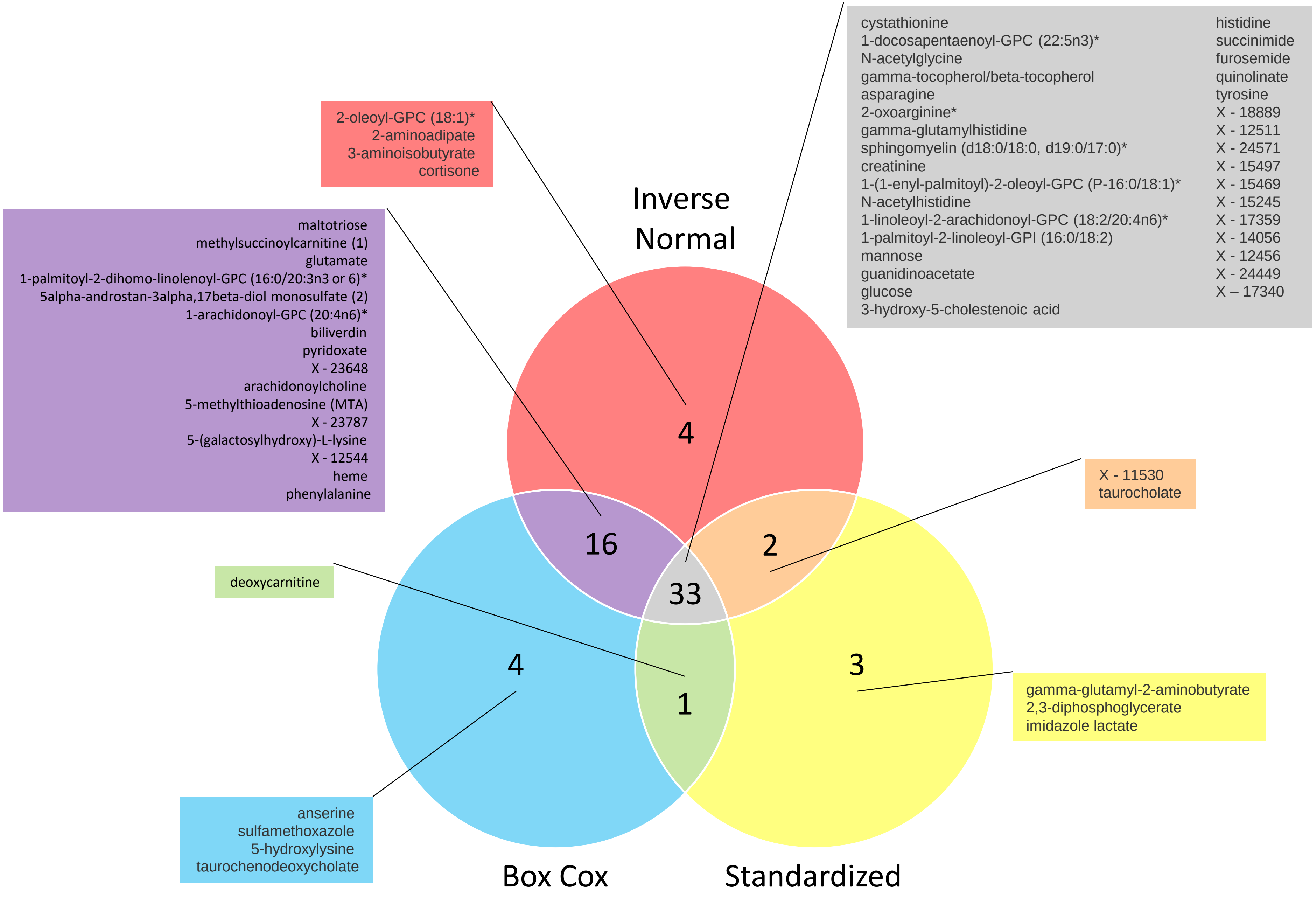

Supplementary Figure 7. Elastic Net selection by metabolite transformation in MA for HOMA-IR.

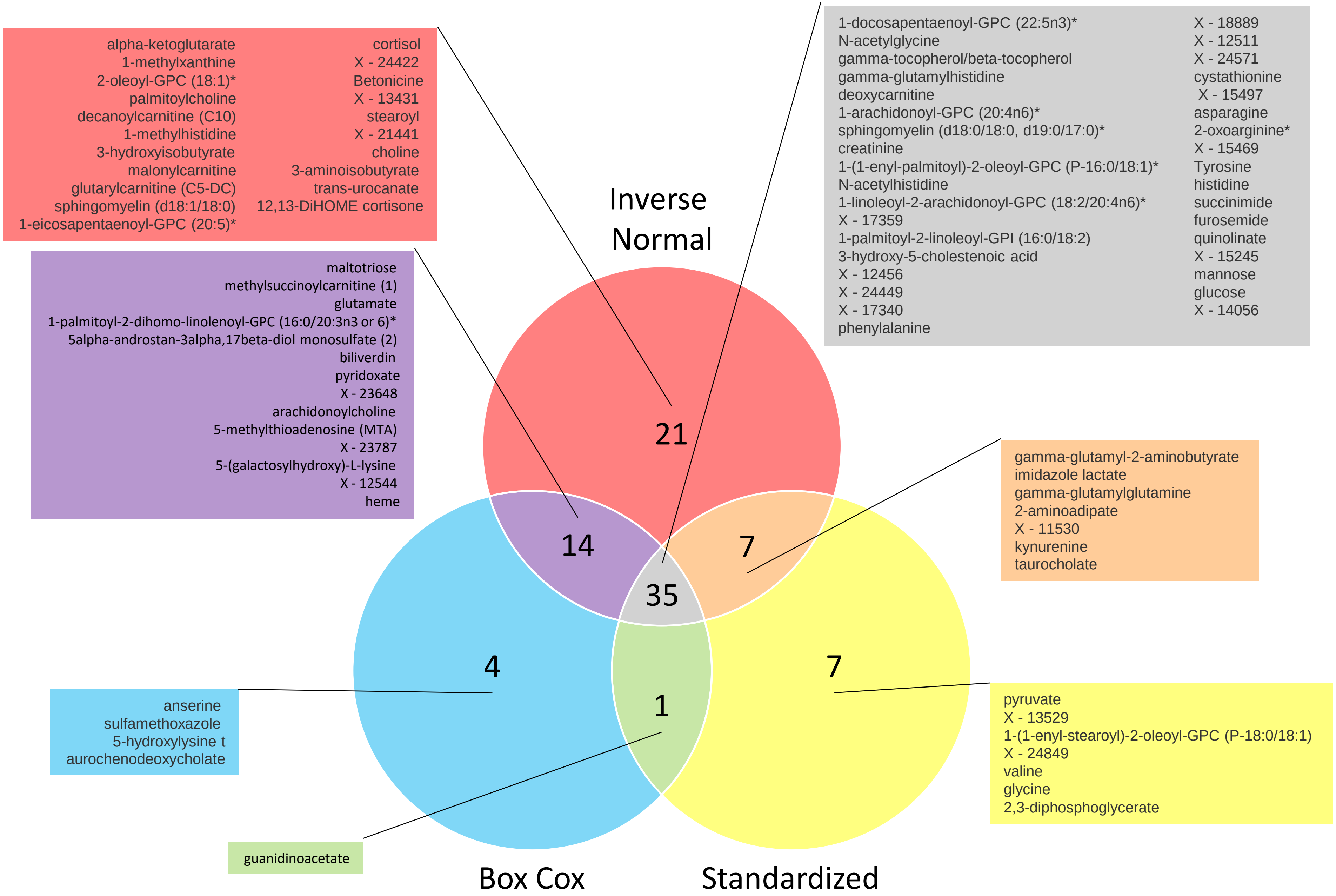

Supplement: Supplementary file 1 — Supplementary material 1 (PDF 354.4 kb) [file 11306_2023_2035_MOESM1_ESM.pdf]
